# Data set and model code on the optimal operating state of a negative emission polygeneration system

**DOI:** 10.1016/j.dib.2020.105140

**Published:** 2020-01-29

**Authors:** Kathleen B. Aviso, Raymond R. Tan, Dominic C.Y. Foo, Jui-Yuan Lee, Aristotle T. Ubando

**Affiliations:** a Chemical Engineering Department, De La Salle University, 2401 Taft Avenue, 0922 Manila, Philippines; b Department Chemical and Environmental Engineering/Centre of Excellence for Green Technologies, University of Nottingham Malaysia, Jalan Broga Road, 43500 Semenyih, Selangor, Malaysia; c Department of Chemical Engineering and Biotechnology, National Taipei University of Technology, 1, Sec. 3, Zhongxiao E. Rd., Taipei 10608, Taiwan, ROC; d Mechanical Engineering Department, De La Salle University, 2401 Taft Avenue, 0922 Manila, Philippines

**Keywords:** Negative emissions technology, Carbon dioxide removal, Polygeneration, Desalination, Carbon tax

## Abstract

This article contains the data set and model code for the negative emission polygeneration system described in Tan et al. (2019). The data was generated utilizing an optimization model implemented in LINGO 18.0 and includes information on the operating state of each process unit in the system. The maximum annual profit of the system was determined at different carbon footprint targets. The data set and model code can be utilized for further analysis on the interdependence between the process units of this polygeneration system, its operational and environmental performance, and the potential impact of integrating new process units into the network.

**Table:** Specifications Table

Subject	Renewable Energy, Sustainability and Environment
Specific subject area	Optimal operating state of a negative emission polygeneration system
Type of data	Numerical data obtained from optimizing the system presented in Tan et al. (2019) using the code in the supplementary file under different carbon footprint targets. Data are presented in tabular and graphical form.
How data were acquired	The data presented were obtained from the results of an optimization model which was implemented in LINGO 18.0 using a laptop with processor Intel®Core™ i7-6500U @ 2.50 GHz with 8.00 GB RAM.
Data format	Raw, Processed
Parameters for data collection	The parameters needed include the price for electricity, heat, cooling, water and hydrochloric acid and capital costs for process and storage units of the polygeneration system.
Description of data collection	Given 11 carbon footprint targets, data regarding the maximum annual profit that the system can achieve and the operating states of the process and storage units are gathered. Data were generated automatically using the model described in Tan et al. (2019) which was coded in LINGO 18.0 and found in Appendix A
Data source location	Data is in this article.
Data accessibility	All data is in this article or can be generated using the code given here. The supplementary file contains the data
Related research article	Author's name: Raymond R. Tan, Kathleen B. Aviso, Dominic C. Y. Foo, Jui-Yuan Lee, Aristotle T. Ubando Title: Optimal synthesis of negative emissions polygeneration systems with desalination Journal: Energy https://doi.org/10.1016/j.energy.2019.115953

**Table:** 

**Value of the Data** • Contains additional scenarios which summarize the trade-off between profitability and carbon footprint. • Useful for researchers looking to extend the application of negative emission polygeneration systems. • Data can be used for developing policies for carbon tax. • Contains the computer code used to generate data in Tan et al. (2019)

## 1 Data

This data article presents the different optimal operating states of a negative emission polygeneration system (NEPS) described in Tan et al. [1] under different scenarios. The NEPS considered here integrates the process proposed by Davies et al. [2] into a multi-product system. The input parameters include the price of electricity, heat, cooling, water and hydrochloric acid (HCl), the capital costs for the process and storage units and the target carbon footprint for the system.

Relevant data are organized as follows. Table 1 gives the mass and energy balance data of the process units. Table 2 gives the prices of streams, while Table 3, Table 4 give the capital costs for the process units and the storage units, respectively. Table 5 gives hourly variations in the demand for electricity, heat, cooling, water and HCl from the NEPs. The price of electricity also changes during the 24-h period and is given in Table 6.

**Table 1:** Material and energy balance data for process units [1].

	Utility Boiler	CHP Unit^a^	Chiller	RO Unit	EGDA
Biomass fuel (kg)	−0.25	−0.80			
Electricity (kWh)		+1	−0.2	−3	−0.013
Steam (kWh)	+1	+1.6			
Cooling (kWh)			1		
Purified water (t)	−0.002	−0.003		+1	−0.1
HCl (t)					+0.1
Seawater (t)				−2	
Brine (t)				+1	−1
Treated brine (t)					+1

a Externally fired gas turbine (EFGT) with heat recovery steam generator (HRSG).

**Table 2:** Price of streams [1].

	Price Range (€ per unit)
Biomass fuel (kg)	0.20
Electricity (kWh)^a^	0–0.12
Steam (kWh)	0.04
Cooling (kWh)	0.06
Purified water (t)	1.20
HCl (t)	80.00
Seawater (t)	0
Brine (t)	0
Treated brine (t)	0

a Price varies within a 24-h cycle.

**Table 3:** Capital costs of process units and associated part-load limits [1].

	Utility Boiler	CHP Unit	Chiller	RO Unit	Electrolysis/GDA
Fixed Cost Component	€ 45,000	€ 380,000	€ 44,000	0	0
Variable Cost Component	€ 175/kW	€ 950/kW	€ 268/kW	€ 15,000/t	€ 350/t
Part-load limit coefficients	0.30	0.30	0.25	0	0

^a^For sensitivity analysis.

**Table 4:** Capital costs for storage units [1].

	Purified Water	HCl or Brine
Fixed Cost Component	€ 16,000	€ 40,000
Variable Cost Component	€ 150/m^3^	€ 375/m^3^

**Table 5:** Hourly demands for electricity, heat, cooling, water and HCl.

Period	Electricity	Heat	Cooling	Water	HCl
1	4000	12,000	0	100	8
2	4000	12,000	0	100	8
3	4000	12,000	0	100	8
4	4000	12,000	0	100	8
5	6000	12,000	0	100	8
6	6000	12,000	0	100	8
7	6000	8000	0	100	8
8	8000	8000	0	100	8
9	8000	8000	1000	100	8
10	10,000	4000	1000	100	8
11	10,000	4000	1500	100	8
12	10,000	4000	1500	100	8
13	10,000	4000	1500	100	8
14	8000	4000	1500	100	8
15	8000	4000	1500	100	8
16	8000	8000	1500	100	8
17	8000	8000	1000	100	8
18	8000	8000	1000	100	8
19	10,000	10,000	500	100	8
20	10,000	10,000	500	100	8
21	10,000	10,000	0	100	8
22	4000	12,000	0	100	8
23	4000	12,000	0	100	8
24	4000	12,000	0	100	8

**Table 6:** Electricity price variations during the 24-h period.

Period	Price of electricity (€/kWh)
1	0.050
2	0.030
3	0.020
4	0.040
5	0.050
6	0.050
7	0.080
8	0.080
9	0.100
10	0.120
11	0.120
12	0.100
13	0.090
14	0.070
15	0.070
16	0.060
17	0.080
18	0.080
19	0.090
20	0.100
21	0.110
22	0.080
23	0.070
24	0.060

**Fig. 1:**
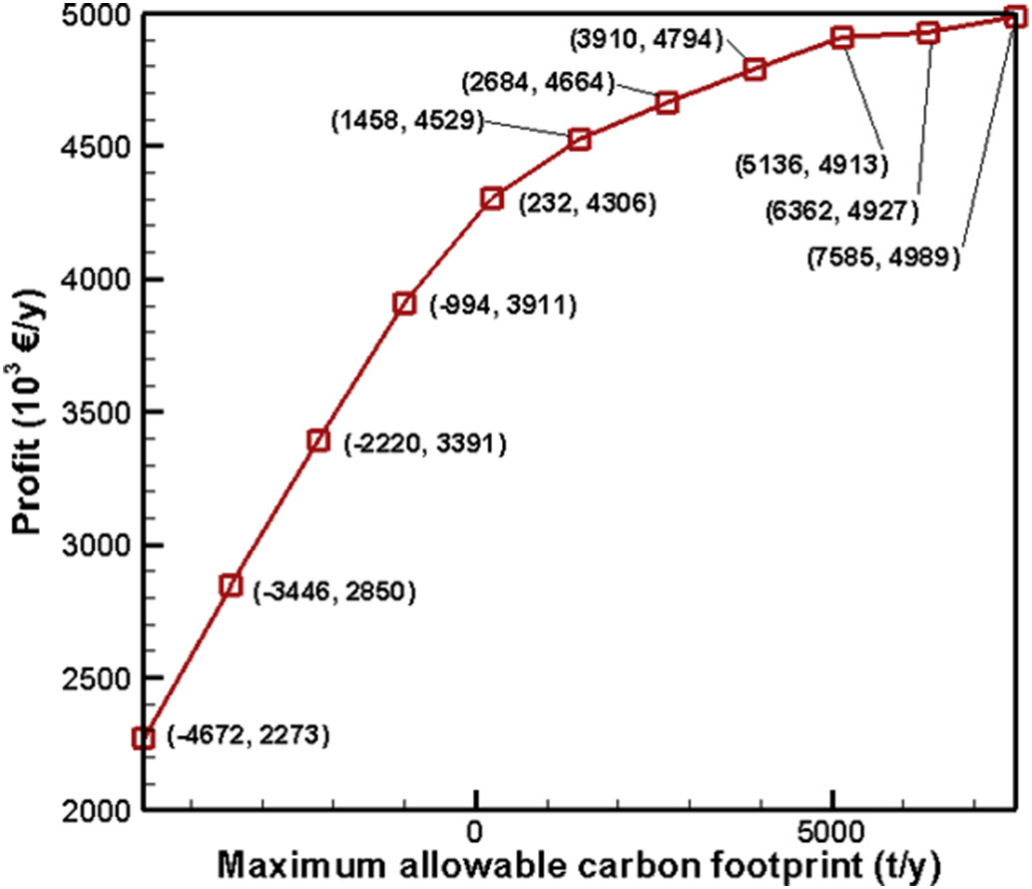
Trade-off between carbon footprint and annual profit.

**Fig. 2:**
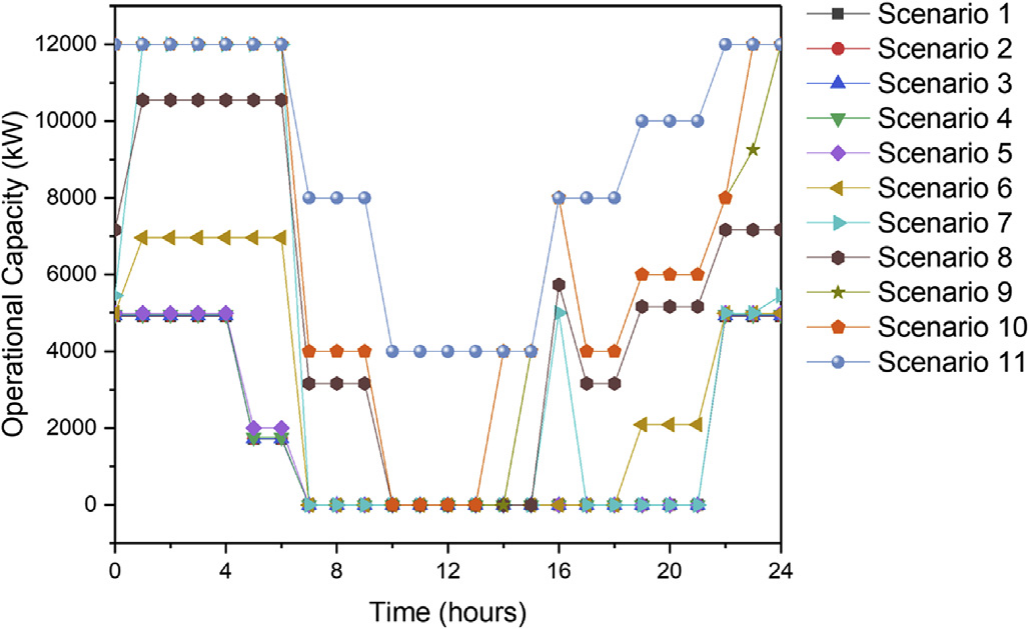
Optimal operating state of the boiler for all scenarios.

**Fig. 3:**
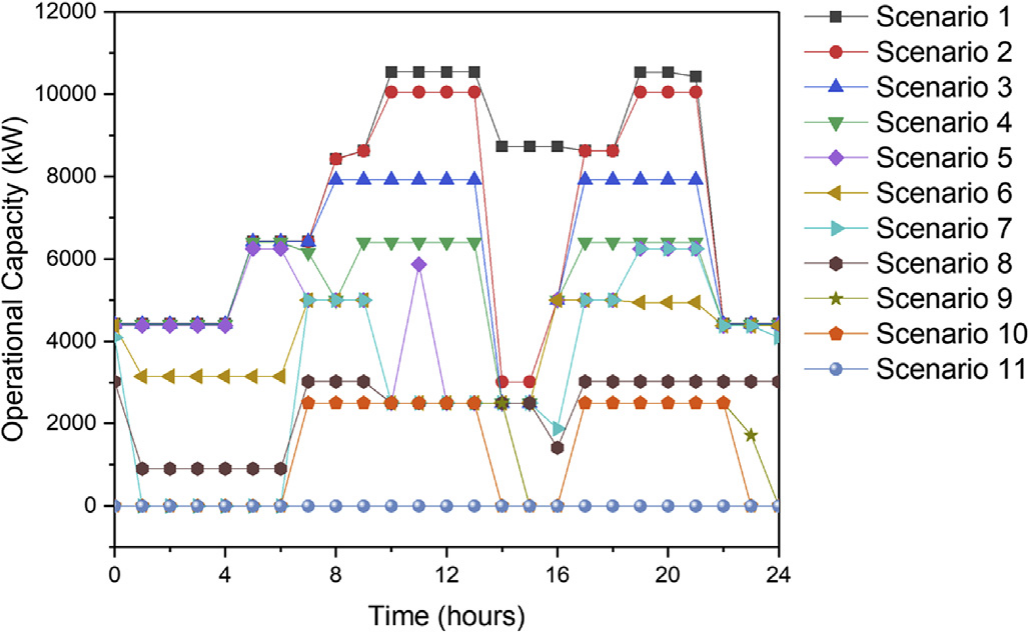
Optimal operating state of the CHP for all scenarios.

**Fig. 4:**
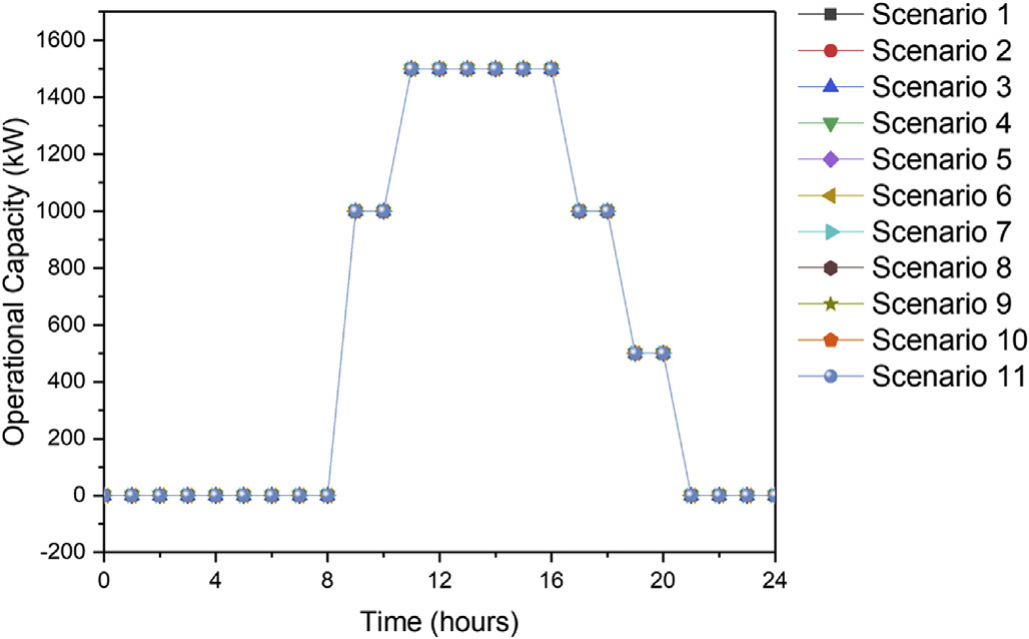
Optimal operating state of the chiller for all scenarios.

**Fig. 5:**
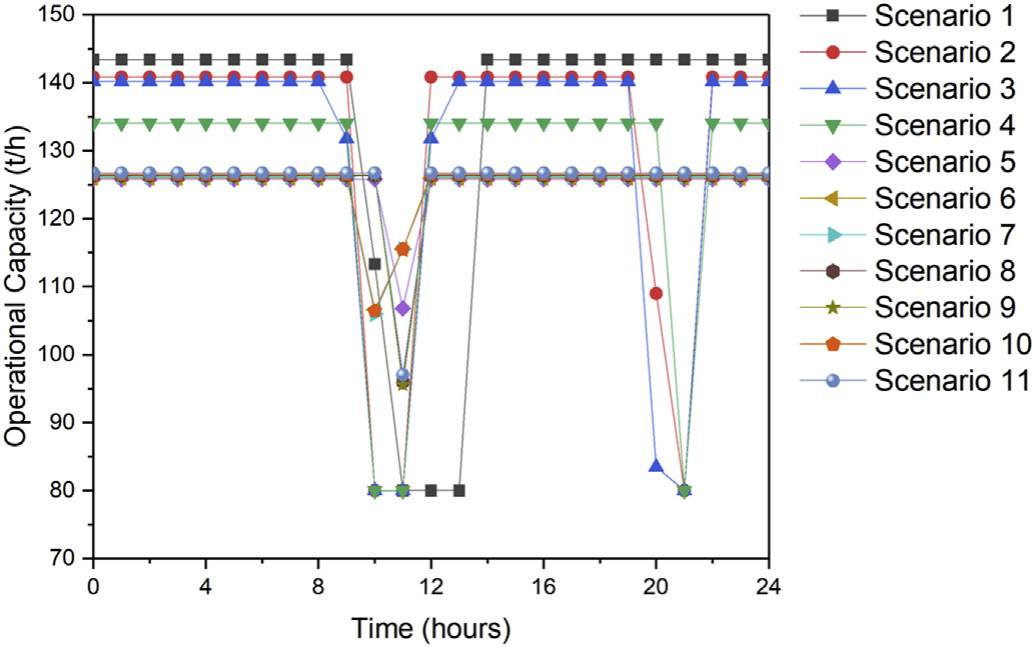
Optimal operating state of the RO for all scenarios.

**Fig. 6:**
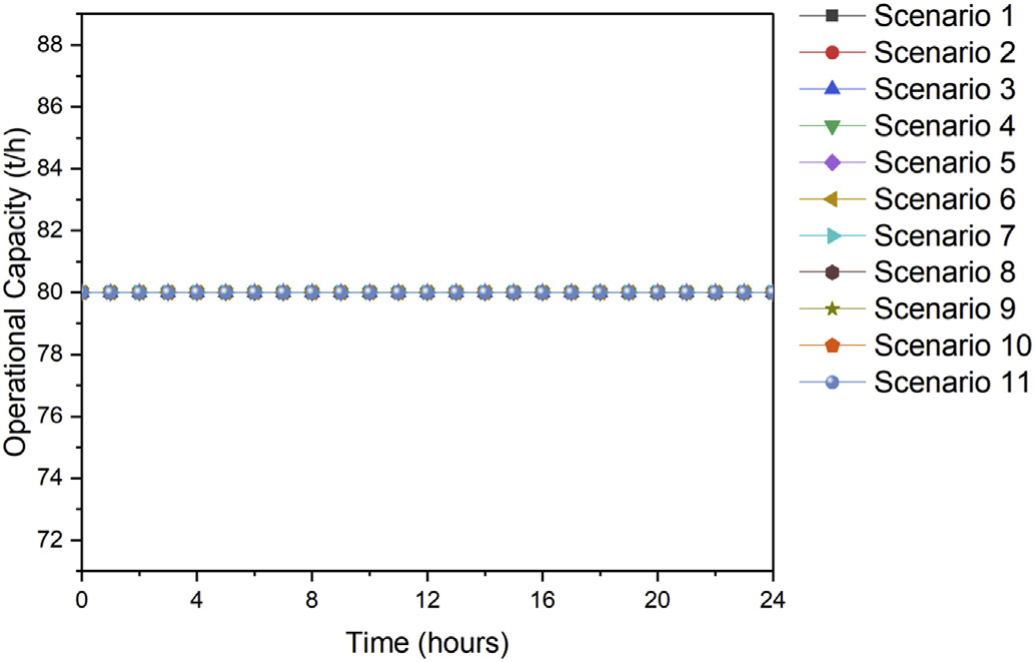
Optimal operating state of the EGDA for all scenarios.

**Fig. 7:**
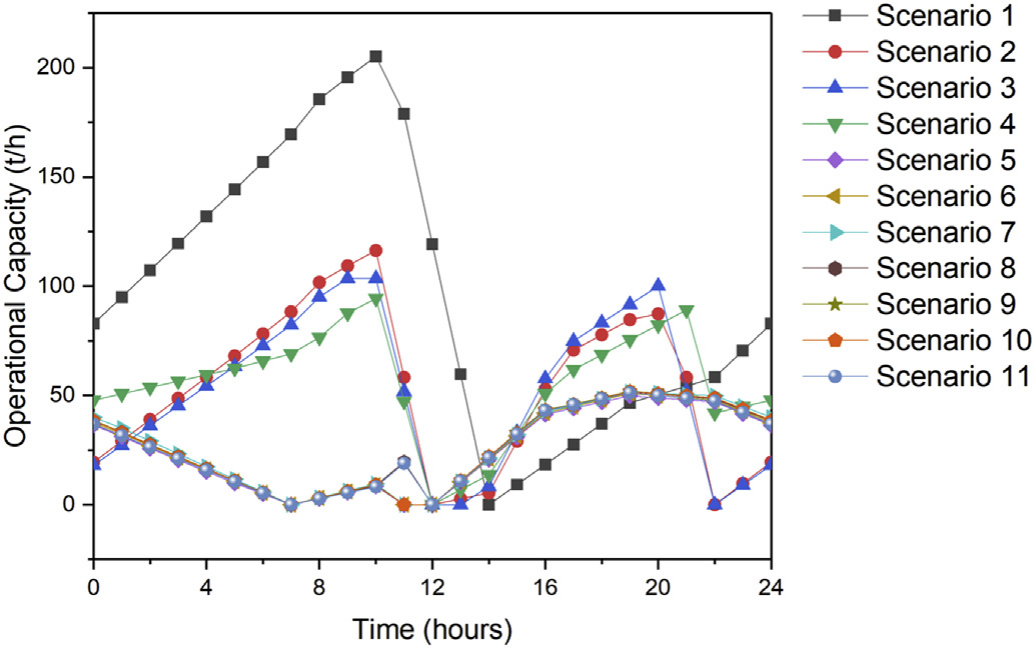
Optimal operating state of the water storage for all scenarios.

Fig. 1 contains the summary of the trade-off between annual profit and carbon footprint of the different scenarios. Fig. 2, Fig. 3, Fig. 4, Fig. 5, Fig. 6 contain the optimal operating state for the boiler, CHP, chiller, RO and EGDA units. Fig. 7 contains the optimal capacity of the water storage unit. The Supplementary File contains the model code and the data used to generate Fig. 1, Fig. 2, Fig. 3, Fig. 4, Fig. 5, Fig. 6, Fig. 7. The Supplementary Excel File contains the Input Data used by the model code.

## 2 Experimental design, materials and method

The data is used to identify the optimal operating state of the NEPS using the mixed-integer linear optimization model described in Tan et al. [1] and executed in LINGO 18.0 [3] which can be downloaded from www.lindo.com. The code can be found in the supplementary file. The annual profit is maximized in consideration of 11 different scenarios, which varied only in the target carbon footprint. Furthermore, it is assumed that electricity price varies per hour; the EGDA variable cost is € 350/kW; the price of treated brine is € 0/t (corresponding to no price for CO_2_ captured); and that the price of HCl is € 80/t. The first scenario maximizes the annual profit with the maximum target carbon footprint of −4671.95 t/y, which is the lowest possible carbon footprint that can be achieved under these conditions. The last scenario maximizes the annual profit with a maximum target carbon footprint of 7585.32 t/y, which corresponds to the highest possible carbon footprint achieved under the given conditions. Scenarios 2 to 10 maximized the annual profit under different carbon footprint targets which have been generated by dividing the CO_2_ range into 10 increments. The summary of the trade-off between annual profit and carbon footprint is summarized in Fig. 1. The optimal operating state for the boiler, CHP, chiller, RO, and EGDA are shown in Fig. 2, Fig. 3, Fig. 4, Fig. 5, Fig. 6, respectively. The optimal capacity usage of the water storage capacity is shown in Fig. 7. The results shown in Fig. 2, Fig. 3, Fig. 4, Fig. 5, Fig. 6, Fig. 7 highlight the comparison of the 11 scenarios. The data used to generate Fig. 1, Fig. 2, Fig. 3, Fig. 4, Fig. 5, Fig. 6, Fig. 7 are also provided in the Supplementary file.
