# Supplementary material for: Data set and model code on the optimal operating state of a negative emission polygeneration system
Source: Data Brief. 2020 Jan 29;29:105140. doi: 10.1016/j.dib.2020.105140 (PMC7021537; doi:10.1016/j.dib.2020.105140)
Supplement: Multimedia component 1 [file mmc1.docx]

Supplementary File

This file contains the data used to generate Figures 1 to 7 in the manuscript.

Table A.1. Summary of trade-off between annual profit and carbon footprint for the different scenarios

| Scenario | Annual Profit  (in Thousand €/y) | Carbon Footprint  (in t/y) |
| --- | --- | --- |
| 1 | 2,273.17 | (4,671.95) |
| 2 | 2,850.20 | (3,445.95) |
| 3 | 3,390.72 | (2,219.95) |
| 4 | 3,910.62 | (993.95) |
| 5 | 4,306.37 | 232.05 |
| 6 | 4,529.29 | 1,458.05 |
| 7 | 4,663.97 | 2,684.05 |
| 8 | 4,793.60 | 3,910.05 |
| 9 | 4,913.37 | 5,136.05 |
| 10 | 4,927.32 | 6,362.05 |
| 11 | 4,988.59 | 7,585.32 |

Table A.2 The resulting operational capacity of the boiler (in kW) for all scenarios. (Data for Figure 2)

| Time (hours) | Scenario 1 | Scenario 2 | Scenario 3 | Scenario 4 | Scenario 5 | Scenario 6 | Scenario 7 | Scenario 8 | Scenario 9 | Scenario 10 | Scenario 11 |
| --- | --- | --- | --- | --- | --- | --- | --- | --- | --- | --- | --- |
| 0 | 4910.08 | 4922.35 | 4925.54 | 4954.94 | 4994.39 | 4993.63 | 5453.44 | 7164.84 | 12000.00 | 12000.00 | 12000.00 |
| 1 | 4910.08 | 4922.35 | 4925.54 | 4954.94 | 4994.39 | 6963.96 | 12000.00 | 10549.45 | 12000.00 | 12000.00 | 12000.00 |
| 2 | 4910.08 | 4922.35 | 4925.54 | 4954.94 | 4994.39 | 6963.96 | 12000.00 | 10549.45 | 12000.00 | 12000.00 | 12000.00 |
| 3 | 4910.08 | 4922.35 | 4925.54 | 4954.94 | 4994.39 | 6963.96 | 12000.00 | 10549.45 | 12000.00 | 12000.00 | 12000.00 |
| 4 | 4910.08 | 4922.35 | 4925.54 | 4954.94 | 4994.39 | 6963.96 | 12000.00 | 10549.45 | 12000.00 | 12000.00 | 12000.00 |
| 5 | 1710.08 | 1722.35 | 1725.54 | 1754.94 | 2000.00 | 6963.96 | 12000.00 | 10549.45 | 12000.00 | 12000.00 | 12000.00 |
| 6 | 1710.08 | 1722.35 | 1725.54 | 1754.94 | 2000.00 | 6963.96 | 12000.00 | 10549.45 | 12000.00 | 12000.00 | 12000.00 |
| 7 | 0.00 | 0.00 | 0.00 | 0.00 | 0.00 | 0.00 | 0.00 | 3164.84 | 4000.00 | 4000.00 | 8000.00 |
| 8 | 0.00 | 0.00 | 0.00 | 0.00 | 0.00 | 0.00 | 0.00 | 3164.84 | 4000.00 | 4000.00 | 8000.00 |
| 9 | 0.00 | 0.00 | 0.00 | 0.00 | 0.00 | 0.00 | 0.00 | 3164.84 | 4000.00 | 4000.00 | 8000.00 |
| 10 | 0.00 | 0.00 | 0.00 | 0.00 | 0.00 | 0.00 | 0.00 | 0.00 | 0.00 | 0.00 | 4000.00 |
| 11 | 0.00 | 0.00 | 0.00 | 0.00 | 0.00 | 0.00 | 0.00 | 0.00 | 0.00 | 0.00 | 4000.00 |
| 12 | 0.00 | 0.00 | 0.00 | 0.00 | 0.00 | 0.00 | 0.00 | 0.00 | 0.00 | 0.00 | 4000.00 |
| 13 | 0.00 | 0.00 | 0.00 | 0.00 | 0.00 | 0.00 | 0.00 | 0.00 | 0.00 | 0.00 | 4000.00 |
| 14 | 0.00 | 0.00 | 0.00 | 0.00 | 0.00 | 0.00 | 0.00 | 0.00 | 0.00 | 4000.00 | 4000.00 |
| 15 | 0.00 | 0.00 | 0.00 | 0.00 | 0.00 | 0.00 | 0.00 | 0.00 | 4000.00 | 4000.00 | 4000.00 |
| 16 | 0.00 | 0.00 | 0.00 | 0.00 | 0.00 | 0.00 | 5000.00 | 5735.61 | 8000.00 | 8000.00 | 8000.00 |
| 17 | 0.00 | 0.00 | 0.00 | 0.00 | 0.00 | 0.00 | 0.00 | 3164.84 | 4000.00 | 4000.00 | 8000.00 |
| 18 | 0.00 | 0.00 | 0.00 | 0.00 | 0.00 | 0.00 | 0.00 | 3164.84 | 4000.00 | 4000.00 | 8000.00 |
| 19 | 0.00 | 0.00 | 0.00 | 0.00 | 0.00 | 2089.19 | 0.00 | 5164.84 | 6000.00 | 6000.00 | 10000.00 |
| 20 | 0.00 | 0.00 | 0.00 | 0.00 | 0.00 | 2089.19 | 0.00 | 5164.84 | 6000.00 | 6000.00 | 10000.00 |
| 21 | 0.00 | 0.00 | 0.00 | 0.00 | 0.00 | 2089.19 | 0.00 | 5164.84 | 6000.00 | 6000.00 | 10000.00 |
| 22 | 4910.08 | 4922.35 | 4925.54 | 4954.94 | 4994.39 | 4993.63 | 4992.70 | 7164.84 | 8000.00 | 8000.00 | 12000.00 |
| 23 | 4910.08 | 4922.35 | 4925.54 | 4954.94 | 4994.39 | 4993.63 | 4992.70 | 7164.84 | 9252.15 | 12000.00 | 12000.00 |
| 24 | 4910.08 | 4922.35 | 4925.54 | 4954.94 | 4994.39 | 4993.63 | 5453.44 | 7164.84 | 12000.00 | 12000.00 | 12000.00 |

Table A.3 The resulting operational capacity of the CHP (in kW) for all scenarios. (Data for Figure 3)

| Time (hours) | Scenario 1 | Scenario 2 | Scenario 3 | Scenario 4 | Scenario 5 | Scenario 6 | Scenario 7 | Scenario 8 | Scenario 9 | Scenario 10 | Scenario 11 |
| --- | --- | --- | --- | --- | --- | --- | --- | --- | --- | --- | --- |
| 0 | 4431.20 | 4423.53 | 4421.54 | 4403.16 | 4378.51 | 4378.98 | 4091.60 | 3021.98 | 0.00 | 0.00 | 0.00 |
| 1 | 4431.20 | 4423.53 | 4421.54 | 4403.16 | 4378.51 | 3147.53 | 0.00 | 906.59 | 0.00 | 0.00 | 0.00 |
| 2 | 4431.20 | 4423.53 | 4421.54 | 4403.16 | 4378.51 | 3147.53 | 0.00 | 906.59 | 0.00 | 0.00 | 0.00 |
| 3 | 4431.20 | 4423.53 | 4421.54 | 4403.16 | 4378.51 | 3147.53 | 0.00 | 906.59 | 0.00 | 0.00 | 0.00 |
| 4 | 4431.20 | 4423.53 | 4421.54 | 4403.16 | 4378.51 | 3147.53 | 0.00 | 906.59 | 0.00 | 0.00 | 0.00 |
| 5 | 6431.20 | 6423.53 | 6421.54 | 6403.16 | 6250.00 | 3147.53 | 0.00 | 906.59 | 0.00 | 0.00 | 0.00 |
| 6 | 6431.20 | 6423.53 | 6421.54 | 6403.16 | 6250.00 | 3147.53 | 0.00 | 906.59 | 0.00 | 0.00 | 0.00 |
| 7 | 6431.20 | 6423.53 | 6421.54 | 6158.84 | 5000.00 | 5000.00 | 5000.00 | 3021.98 | 2500.00 | 2500.00 | 0.00 |
| 8 | 8431.20 | 8423.53 | 7923.56 | 5000.00 | 5000.00 | 5000.00 | 5000.00 | 3021.98 | 2500.00 | 2500.00 | 0.00 |
| 9 | 8631.20 | 8623.53 | 7923.56 | 6403.16 | 5000.00 | 5000.00 | 5000.00 | 3021.98 | 2500.00 | 2500.00 | 0.00 |
| 10 | 10541.02 | 10052.90 | 7923.56 | 6403.16 | 2500.00 | 2500.00 | 2500.00 | 2500.00 | 2500.00 | 2500.00 | 0.00 |
| 11 | 10541.02 | 10052.90 | 7923.56 | 6403.16 | 5866.79 | 2500.00 | 2500.00 | 2500.00 | 2500.00 | 2500.00 | 0.00 |
| 12 | 10541.02 | 10052.90 | 7923.56 | 6403.16 | 2500.00 | 2500.00 | 2500.00 | 2500.00 | 2500.00 | 2500.00 | 0.00 |
| 13 | 10541.02 | 10052.90 | 7923.56 | 6403.16 | 2500.00 | 2500.00 | 2500.00 | 2500.00 | 2500.00 | 2500.00 | 0.00 |
| 14 | 8731.20 | 3015.87 | 2500.00 | 2500.00 | 2500.00 | 2500.00 | 2500.00 | 2500.00 | 2500.00 | 0.00 | 0.00 |
| 15 | 8731.20 | 3015.87 | 2500.00 | 2500.00 | 2500.00 | 2500.00 | 2500.00 | 2500.00 | 0.00 | 0.00 | 0.00 |
| 16 | 8731.20 | 5000.00 | 5000.00 | 5000.00 | 5000.00 | 5000.00 | 1875.00 | 1415.25 | 0.00 | 0.00 | 0.00 |
| 17 | 8631.20 | 8623.53 | 7923.56 | 6403.16 | 5000.00 | 5000.00 | 5000.00 | 3021.98 | 2500.00 | 2500.00 | 0.00 |
| 18 | 8631.20 | 8623.53 | 7923.56 | 6403.16 | 5000.00 | 5000.00 | 5000.00 | 3021.98 | 2500.00 | 2500.00 | 0.00 |
| 19 | 10531.20 | 10052.90 | 7923.56 | 6403.16 | 6250.00 | 4944.26 | 6250.00 | 3021.98 | 2500.00 | 2500.00 | 0.00 |
| 20 | 10531.20 | 10052.90 | 7923.56 | 6403.16 | 6250.00 | 4944.26 | 6250.00 | 3021.98 | 2500.00 | 2500.00 | 0.00 |
| 21 | 10431.20 | 10052.90 | 7923.56 | 6403.16 | 6250.00 | 4944.26 | 6250.00 | 3021.98 | 2500.00 | 2500.00 | 0.00 |
| 22 | 4431.20 | 4423.53 | 4421.54 | 4403.16 | 4378.51 | 4378.98 | 4379.56 | 3021.98 | 2500.00 | 2500.00 | 0.00 |
| 23 | 4431.20 | 4423.53 | 4421.54 | 4403.16 | 4378.51 | 4378.98 | 4379.56 | 3021.98 | 1717.41 | 0.00 | 0.00 |
| 24 | 4431.20 | 4423.53 | 4421.54 | 4403.16 | 4378.51 | 4378.98 | 4091.60 | 3021.98 | 0.00 | 0.00 | 0.00 |

Table A.4 The resulting operational capacity of the Chiller (in kW) for all scenarios. (Data for Figure 4)

| Time (hours) | Scenario 1 | Scenario 2 | Scenario 3 | Scenario 4 | Scenario 5 | Scenario 6 | Scenario 7 | Scenario 8 | Scenario 9 | Scenario 10 | Scenario 11 |
| --- | --- | --- | --- | --- | --- | --- | --- | --- | --- | --- | --- |
| 0 | 0.00 | 0.00 | 0.00 | 0.00 | 0.00 | 0.00 | 0.00 | 0.00 | 0.00 | 0.00 | 0.00 |
| 1 | 0.00 | 0.00 | 0.00 | 0.00 | 0.00 | 0.00 | 0.00 | 0.00 | 0.00 | 0.00 | 0.00 |
| 2 | 0.00 | 0.00 | 0.00 | 0.00 | 0.00 | 0.00 | 0.00 | 0.00 | 0.00 | 0.00 | 0.00 |
| 3 | 0.00 | 0.00 | 0.00 | 0.00 | 0.00 | 0.00 | 0.00 | 0.00 | 0.00 | 0.00 | 0.00 |
| 4 | 0.00 | 0.00 | 0.00 | 0.00 | 0.00 | 0.00 | 0.00 | 0.00 | 0.00 | 0.00 | 0.00 |
| 5 | 0.00 | 0.00 | 0.00 | 0.00 | 0.00 | 0.00 | 0.00 | 0.00 | 0.00 | 0.00 | 0.00 |
| 6 | 0.00 | 0.00 | 0.00 | 0.00 | 0.00 | 0.00 | 0.00 | 0.00 | 0.00 | 0.00 | 0.00 |
| 7 | 0.00 | 0.00 | 0.00 | 0.00 | 0.00 | 0.00 | 0.00 | 0.00 | 0.00 | 0.00 | 0.00 |
| 8 | 0.00 | 0.00 | 0.00 | 0.00 | 0.00 | 0.00 | 0.00 | 0.00 | 0.00 | 0.00 | 0.00 |
| 9 | 1000.00 | 1000.00 | 1000.00 | 1000.00 | 1000.00 | 1000.00 | 1000.00 | 1000.00 | 1000.00 | 1000.00 | 1000.00 |
| 10 | 1000.00 | 1000.00 | 1000.00 | 1000.00 | 1000.00 | 1000.00 | 1000.00 | 1000.00 | 1000.00 | 1000.00 | 1000.00 |
| 11 | 1500.00 | 1500.00 | 1500.00 | 1500.00 | 1500.00 | 1500.00 | 1500.00 | 1500.00 | 1500.00 | 1500.00 | 1500.00 |
| 12 | 1500.00 | 1500.00 | 1500.00 | 1500.00 | 1500.00 | 1500.00 | 1500.00 | 1500.00 | 1500.00 | 1500.00 | 1500.00 |
| 13 | 1500.00 | 1500.00 | 1500.00 | 1500.00 | 1500.00 | 1500.00 | 1500.00 | 1500.00 | 1500.00 | 1500.00 | 1500.00 |
| 14 | 1500.00 | 1500.00 | 1500.00 | 1500.00 | 1500.00 | 1500.00 | 1500.00 | 1500.00 | 1500.00 | 1500.00 | 1500.00 |
| 15 | 1500.00 | 1500.00 | 1500.00 | 1500.00 | 1500.00 | 1500.00 | 1500.00 | 1500.00 | 1500.00 | 1500.00 | 1500.00 |
| 16 | 1500.00 | 1500.00 | 1500.00 | 1500.00 | 1500.00 | 1500.00 | 1500.00 | 1500.00 | 1500.00 | 1500.00 | 1500.00 |
| 17 | 1000.00 | 1000.00 | 1000.00 | 1000.00 | 1000.00 | 1000.00 | 1000.00 | 1000.00 | 1000.00 | 1000.00 | 1000.00 |
| 18 | 1000.00 | 1000.00 | 1000.00 | 1000.00 | 1000.00 | 1000.00 | 1000.00 | 1000.00 | 1000.00 | 1000.00 | 1000.00 |
| 19 | 500.00 | 500.00 | 500.00 | 500.00 | 500.00 | 500.00 | 500.00 | 500.00 | 500.00 | 500.00 | 500.00 |
| 20 | 500.00 | 500.00 | 500.00 | 500.00 | 500.00 | 500.00 | 500.00 | 500.00 | 500.00 | 500.00 | 500.00 |
| 21 | 0.00 | 0.00 | 0.00 | 0.00 | 0.00 | 0.00 | 0.00 | 0.00 | 0.00 | 0.00 | 0.00 |
| 22 | 0.00 | 0.00 | 0.00 | 0.00 | 0.00 | 0.00 | 0.00 | 0.00 | 0.00 | 0.00 | 0.00 |
| 23 | 0.00 | 0.00 | 0.00 | 0.00 | 0.00 | 0.00 | 0.00 | 0.00 | 0.00 | 0.00 | 0.00 |
| 24 | 0.00 | 0.00 | 0.00 | 0.00 | 0.00 | 0.00 | 0.00 | 0.00 | 0.00 | 0.00 | 0.00 |

Table A.5 The resulting operational capacity of the RO (in tons) for all scenarios. (Data for Figure 5)

| Time (hours) | Scenario 1 | Scenario 2 | Scenario 3 | Scenario 4 | Scenario 5 | Scenario 6 | Scenario 7 | Scenario 8 | Scenario 9 | Scenario 10 | Scenario 11 |
| --- | --- | --- | --- | --- | --- | --- | --- | --- | --- | --- | --- |
| 0 | 143.39 | 140.83 | 140.17 | 134.04 | 125.82 | 125.98 | 126.17 | 126.30 | 126.48 | 126.53 | 126.74 |
| 1 | 143.39 | 140.83 | 140.17 | 134.04 | 125.82 | 125.98 | 126.17 | 126.30 | 126.48 | 126.53 | 126.74 |
| 2 | 143.39 | 140.83 | 140.17 | 134.04 | 125.82 | 125.98 | 126.17 | 126.30 | 126.48 | 126.53 | 126.74 |
| 3 | 143.39 | 140.83 | 140.17 | 134.04 | 125.82 | 125.98 | 126.17 | 126.30 | 126.48 | 126.53 | 126.74 |
| 4 | 143.39 | 140.83 | 140.17 | 134.04 | 125.82 | 125.98 | 126.17 | 126.30 | 126.48 | 126.53 | 126.74 |
| 5 | 143.39 | 140.83 | 140.17 | 134.04 | 125.82 | 125.98 | 126.17 | 126.30 | 126.48 | 126.53 | 126.74 |
| 6 | 143.39 | 140.83 | 140.17 | 134.04 | 125.82 | 125.98 | 126.17 | 126.30 | 126.48 | 126.53 | 126.74 |
| 7 | 143.39 | 140.83 | 140.17 | 134.04 | 125.82 | 125.98 | 126.17 | 126.30 | 126.48 | 126.53 | 126.74 |
| 8 | 143.39 | 140.83 | 140.17 | 134.04 | 125.82 | 125.98 | 126.17 | 126.30 | 126.48 | 126.53 | 126.74 |
| 9 | 143.39 | 140.83 | 131.77 | 134.04 | 125.82 | 125.98 | 126.17 | 126.30 | 126.48 | 126.53 | 126.74 |
| 10 | 113.33 | 80.00 | 80.00 | 80.00 | 125.82 | 106.56 | 105.98 | 126.30 | 126.48 | 106.42 | 126.74 |
| 11 | 80.00 | 80.00 | 80.00 | 80.00 | 106.81 | 115.50 | 115.50 | 95.97 | 95.57 | 115.50 | 97.05 |
| 12 | 80.00 | 140.83 | 131.77 | 134.04 | 125.82 | 125.98 | 126.17 | 126.30 | 126.48 | 126.53 | 126.74 |
| 13 | 80.00 | 140.83 | 140.17 | 134.04 | 125.82 | 125.98 | 126.17 | 126.30 | 126.48 | 126.53 | 126.74 |
| 14 | 143.39 | 140.83 | 140.17 | 134.04 | 125.82 | 125.98 | 126.17 | 126.30 | 126.48 | 126.53 | 126.74 |
| 15 | 143.39 | 140.83 | 140.17 | 134.04 | 125.82 | 125.98 | 126.17 | 126.30 | 126.48 | 126.53 | 126.74 |
| 16 | 143.39 | 140.83 | 140.17 | 134.04 | 125.82 | 125.98 | 126.17 | 126.30 | 126.48 | 126.53 | 126.74 |
| 17 | 143.39 | 140.83 | 140.17 | 134.04 | 125.82 | 125.98 | 126.17 | 126.30 | 126.48 | 126.53 | 126.74 |
| 18 | 143.39 | 140.83 | 140.17 | 134.04 | 125.82 | 125.98 | 126.17 | 126.30 | 126.48 | 126.53 | 126.74 |
| 19 | 143.39 | 140.83 | 140.17 | 134.04 | 125.82 | 125.98 | 126.17 | 126.30 | 126.48 | 126.53 | 126.74 |
| 20 | 143.39 | 108.99 | 83.47 | 134.04 | 125.82 | 125.98 | 126.17 | 126.30 | 126.48 | 126.53 | 126.74 |
| 21 | 143.39 | 80.00 | 80.00 | 80.00 | 125.82 | 125.98 | 126.17 | 126.30 | 126.48 | 126.53 | 126.74 |
| 22 | 143.39 | 140.83 | 140.17 | 134.04 | 125.82 | 125.98 | 126.17 | 126.30 | 126.48 | 126.53 | 126.74 |
| 23 | 143.39 | 140.83 | 140.17 | 134.04 | 125.82 | 125.98 | 126.17 | 126.30 | 126.48 | 126.53 | 126.74 |
| 24 | 143.39 | 140.83 | 140.17 | 134.04 | 125.82 | 125.98 | 126.17 | 126.30 | 126.48 | 126.53 | 126.74 |

Table A.6 The resulting operational capacity of the EGDA (in tons) for all scenarios. (Data for Figure 6)

| Time (hours) | Scenario 1 | Scenario 2 | Scenario 3 | Scenario 4 | Scenario 5 | Scenario 6 | Scenario 7 | Scenario 8 | Scenario 9 | Scenario 10 | Scenario 11 |
| --- | --- | --- | --- | --- | --- | --- | --- | --- | --- | --- | --- |
| 0 | 80.00 | 80.00 | 80.00 | 80.00 | 80.00 | 80.00 | 80.00 | 80.00 | 80.00 | 80.00 | 80.00 |
| 1 | 80.00 | 80.00 | 80.00 | 80.00 | 80.00 | 80.00 | 80.00 | 80.00 | 80.00 | 80.00 | 80.00 |
| 2 | 80.00 | 80.00 | 80.00 | 80.00 | 80.00 | 80.00 | 80.00 | 80.00 | 80.00 | 80.00 | 80.00 |
| 3 | 80.00 | 80.00 | 80.00 | 80.00 | 80.00 | 80.00 | 80.00 | 80.00 | 80.00 | 80.00 | 80.00 |
| 4 | 80.00 | 80.00 | 80.00 | 80.00 | 80.00 | 80.00 | 80.00 | 80.00 | 80.00 | 80.00 | 80.00 |
| 5 | 80.00 | 80.00 | 80.00 | 80.00 | 80.00 | 80.00 | 80.00 | 80.00 | 80.00 | 80.00 | 80.00 |
| 6 | 80.00 | 80.00 | 80.00 | 80.00 | 80.00 | 80.00 | 80.00 | 80.00 | 80.00 | 80.00 | 80.00 |
| 7 | 80.00 | 80.00 | 80.00 | 80.00 | 80.00 | 80.00 | 80.00 | 80.00 | 80.00 | 80.00 | 80.00 |
| 8 | 80.00 | 80.00 | 80.00 | 80.00 | 80.00 | 80.00 | 80.00 | 80.00 | 80.00 | 80.00 | 80.00 |
| 9 | 80.00 | 80.00 | 80.00 | 80.00 | 80.00 | 80.00 | 80.00 | 80.00 | 80.00 | 80.00 | 80.00 |
| 10 | 80.00 | 80.00 | 80.00 | 80.00 | 80.00 | 80.00 | 80.00 | 80.00 | 80.00 | 80.00 | 80.00 |
| 11 | 80.00 | 80.00 | 80.00 | 80.00 | 80.00 | 80.00 | 80.00 | 80.00 | 80.00 | 80.00 | 80.00 |
| 12 | 80.00 | 80.00 | 80.00 | 80.00 | 80.00 | 80.00 | 80.00 | 80.00 | 80.00 | 80.00 | 80.00 |
| 13 | 80.00 | 80.00 | 80.00 | 80.00 | 80.00 | 80.00 | 80.00 | 80.00 | 80.00 | 80.00 | 80.00 |
| 14 | 80.00 | 80.00 | 80.00 | 80.00 | 80.00 | 80.00 | 80.00 | 80.00 | 80.00 | 80.00 | 80.00 |
| 15 | 80.00 | 80.00 | 80.00 | 80.00 | 80.00 | 80.00 | 80.00 | 80.00 | 80.00 | 80.00 | 80.00 |
| 16 | 80.00 | 80.00 | 80.00 | 80.00 | 80.00 | 80.00 | 80.00 | 80.00 | 80.00 | 80.00 | 80.00 |
| 17 | 80.00 | 80.00 | 80.00 | 80.00 | 80.00 | 80.00 | 80.00 | 80.00 | 80.00 | 80.00 | 80.00 |
| 18 | 80.00 | 80.00 | 80.00 | 80.00 | 80.00 | 80.00 | 80.00 | 80.00 | 80.00 | 80.00 | 80.00 |
| 19 | 80.00 | 80.00 | 80.00 | 80.00 | 80.00 | 80.00 | 80.00 | 80.00 | 80.00 | 80.00 | 80.00 |
| 20 | 80.00 | 80.00 | 80.00 | 80.00 | 80.00 | 80.00 | 80.00 | 80.00 | 80.00 | 80.00 | 80.00 |
| 21 | 80.00 | 80.00 | 80.00 | 80.00 | 80.00 | 80.00 | 80.00 | 80.00 | 80.00 | 80.00 | 80.00 |
| 22 | 80.00 | 80.00 | 80.00 | 80.00 | 80.00 | 80.00 | 80.00 | 80.00 | 80.00 | 80.00 | 80.00 |
| 23 | 80.00 | 80.00 | 80.00 | 80.00 | 80.00 | 80.00 | 80.00 | 80.00 | 80.00 | 80.00 | 80.00 |
| 24 | 80.00 | 80.00 | 80.00 | 80.00 | 80.00 | 80.00 | 80.00 | 80.00 | 80.00 | 80.00 | 80.00 |

Table A.7 The resulting operational capacity of the water storage (in tons) for all scenarios. (Data for Figure 7)

| Time (hours) | Scenario 1 | Scenario 2 | Scenario 3 | Scenario 4 | Scenario 5 | Scenario 6 | Scenario 7 | Scenario 8 | Scenario 9 | Scenario 10 | Scenario 11 |
| --- | --- | --- | --- | --- | --- | --- | --- | --- | --- | --- | --- |
| 0 | 82.78 | 19.43 | 18.10 | 47.74 | 36.37 | 37.48 | 39.96 | 38.17 | 38.63 | 38.32 | 36.84 |
| 1 | 95.05 | 29.15 | 27.15 | 50.66 | 31.06 | 32.34 | 34.96 | 33.08 | 33.11 | 32.84 | 31.58 |
| 2 | 107.33 | 38.86 | 36.20 | 53.58 | 25.76 | 26.95 | 29.13 | 27.57 | 27.59 | 27.37 | 26.32 |
| 3 | 119.60 | 48.58 | 45.25 | 56.50 | 20.46 | 21.56 | 23.30 | 22.05 | 22.07 | 21.89 | 21.05 |
| 4 | 131.87 | 58.29 | 54.30 | 59.42 | 15.16 | 16.17 | 17.48 | 16.54 | 16.55 | 16.42 | 15.79 |
| 5 | 144.14 | 68.01 | 63.35 | 62.34 | 9.86 | 10.78 | 11.65 | 11.03 | 11.04 | 10.95 | 10.53 |
| 6 | 156.82 | 78.12 | 72.80 | 65.66 | 4.93 | 5.39 | 5.83 | 5.51 | 5.52 | 5.47 | 5.26 |
| 7 | 169.49 | 88.24 | 82.25 | 68.98 | 0.00 | 0.00 | 0.00 | 0.00 | 0.00 | 0.00 | 0.00 |
| 8 | 185.58 | 101.80 | 95.15 | 76.55 | 2.82 | 2.98 | 3.17 | 2.91 | 2.98 | 3.03 | 2.74 |
| 9 | 195.67 | 109.36 | 103.54 | 87.59 | 5.64 | 5.96 | 6.35 | 5.82 | 5.96 | 6.05 | 5.47 |
| 10 | 205.16 | 116.32 | 103.54 | 94.42 | 8.47 | 8.94 | 9.52 | 8.73 | 8.95 | 9.08 | 8.21 |
| 11 | 178.87 | 58.16 | 51.77 | 47.21 | 0.00 | 0.00 | 0.00 | 19.53 | 0.00 | 0.00 | 18.95 |
| 12 | 119.25 | 0.00 | 0.00 | 0.00 | 0.00 | 0.00 | 0.00 | 0.00 | 0.00 | 0.00 | 0.00 |
| 13 | 59.62 | 2.67 | 0.00 | 6.83 | 10.32 | 10.48 | 10.67 | 10.80 | 10.98 | 11.03 | 10.74 |
| 14 | 0.00 | 5.34 | 8.39 | 13.66 | 20.64 | 20.96 | 21.35 | 21.61 | 21.96 | 22.05 | 21.47 |
| 15 | 9.19 | 29.13 | 33.06 | 32.20 | 30.97 | 31.44 | 32.02 | 32.41 | 32.95 | 32.58 | 32.21 |
| 16 | 18.38 | 52.91 | 57.73 | 50.74 | 41.29 | 41.92 | 42.70 | 43.22 | 43.43 | 43.11 | 42.95 |
| 17 | 27.58 | 70.74 | 74.89 | 61.78 | 44.11 | 44.90 | 45.24 | 45.81 | 45.91 | 45.63 | 45.68 |
| 18 | 37.07 | 77.70 | 83.28 | 68.61 | 46.93 | 47.88 | 48.42 | 48.72 | 48.89 | 48.66 | 48.42 |
| 19 | 46.56 | 84.66 | 91.68 | 75.44 | 49.75 | 50.86 | 51.59 | 51.63 | 51.87 | 51.68 | 51.16 |
| 20 | 50.35 | 87.33 | 100.07 | 82.27 | 48.83 | 49.83 | 51.02 | 50.54 | 50.86 | 50.71 | 49.89 |
| 21 | 54.15 | 58.16 | 51.77 | 89.11 | 47.90 | 48.80 | 50.44 | 49.45 | 49.84 | 49.74 | 48.63 |
| 22 | 58.24 | 0.00 | 0.00 | 41.90 | 46.97 | 47.77 | 49.86 | 48.35 | 48.82 | 48.76 | 47.37 |
| 23 | 70.51 | 9.72 | 9.05 | 44.82 | 41.67 | 42.63 | 44.91 | 43.26 | 43.80 | 43.79 | 42.11 |
| 24 | 82.78 | 19.43 | 18.10 | 47.74 | 36.37 | 37.48 | 39.96 | 38.17 | 38.63 | 38.32 | 36.84 |
