# Supplementary material for: Data set and model code on the optimal operating state of a negative emission polygeneration system
Source: Data Brief. 2020 Jan 29;29:105140. doi: 10.1016/j.dib.2020.105140 (PMC7021537; doi:10.1016/j.dib.2020.105140)
Supplement: Multimedia component 3 [file mmc3.docx]

Model Code

!

PARAMETERS

A - CONTAINS THE TECHNICAL COEFF MATRIX

FC - FIXED COST

P - PRICE PER UNIT STREAM AS A FUNCTION OF PERIOD (IF PRICE VARIES PER PERIOD)

PL - PART LOAD LIMIT FOR PROCESS OPERATION

SFC - FIXED COST FOR STORAGE UNIT

SVC - VARIABLE COST FOR STORAGE UNIT

VC - VARIABLE COST

YT - MINIMUM AMOUNT OF PRODUCT STREAM REQD FOR PRODUCTION

VARIABLES:

B - BINARY VARIABLE FOR SELECTED PROCESSES

BST - BINARY VARIABLE FOR THE ACTIVATION OF STORAGE UNIT

CAPACITY - TOTAL CAPACITY NEEDED FOR STORAGE UNIT

CF - CARBON FOOTPRINT GENERATED PER PERIOD DUE TO ELEC FROM GRID

CO2 - AMOUNT OF CO2 SEQUESTERED AS A FUNCTION OF TREATED BRINE

DIS - AMOUNT GENERATED OF STREAM THAT CAN BE SOLD EXTERNAL TO THE SYSTEM

FSLO - FLOWRATE OF STREAM INTO OR OUT OF STORAGE TANK DURING A PERIOD

SALES - MONETARY INCOME/EXPENSE DUE TO STREAMS

STO - THE VOLUME INSIDE STORAGE TANK DURING A PARTICULAR PERIOD

X - CAPACITY OF PROCESSES

XPARTLOAD - OPERATING CONDITION OF PROCESS DURING A PERIOD

YR - TOTAL AMOUNT OF RAW MATERIAL TO BE SOURCED EXTERNALLY

;

MODEL:

SETS:

PROCESS: FC, VC, X, B, PL;

PERIOD: SALES, CO2, CF, FWATER, FHCL, TIME, OSB,OSCHP, OSCHILL, OSRO, OSGDA, OSELEC, WSTO, HCLSTO,BM,

ELECIMP, ELECPRICE;

STREAM: PP ;

OUTPUT (STREAM);

STORAGE (STREAM): SFC, SVC, CAPACITY, BST;

NOSTORAGE(STREAM);

RM(STREAM): ;

RMREQ (PERIOD, RM): YR;

AMATRIX(STREAM, PROCESS): A;

PMATRIX(PERIOD, STREAM): P;

BMATRIX(PERIOD, PROCESS):XPARTLOAD, SWITCH, Z;

DEMAND(PERIOD, OUTPUT): YT, DIS, STO, FSLO, YI, YH;

ENDSETS

DATA:

PROCESS = BOILER CHP CHILLER RO GDA ;

STREAM = BIOMASS ELEC STEAM COOLING WATER HCL SEAWATER BRINE TRBRINE;

RM = BIOMASS SEAWATER;

OUTPUT = ELEC STEAM COOLING WATER HCL BRINE TRBRINE;

NOSTORAGE = ELEC STEAM COOLING TRBRINE;

STORAGE = WATER HCL BRINE;

PERIOD = 1..24;

!IMPORTS DATA FROM EXCEL FILE “Negative Emission Polygeneration System.xlsx” WHICH IS INCLUDED AS SUPPLEMENTARY MATERIAL, Reference to file address should be updated accordingly;

A, FC, VC, SFC, SVC, YT, P = @OLE('C:\Users\Kathleen Aviso\Documents\Negative Emission Polygeneration System.xlsx', 'A', 'FC', 'VC', 'SFC', 'SVC', 'YT', 'P');

! BOILER CHP CHILLER RO EGDA;

PL = 0.3 0.3 0.25 0 0;

ENDDATA

MAX = ANNUALPROFIT; !objective function for Scenarios 1 to 4;

!MIN = CARBONFOOTPRINT; !objective function for Scenarios 5 and 6;

ANNUALPROFIT = PROFIT - CAPCOST;

CARBONFOOTPRINT < -4671.95;

@FOR(STORAGE(I): @BIN(BST(I))); !BINARY CONSTRAINT ON STORAGE TANKS;

@FOR(PROCESS(J): @BIN(B(J))); !BINARY CONSTRAINT ON PROCESSES;

@FOR(PERIOD(K): @FOR(OUTPUT(I)|I#NE#2: YI(K,I) = 0)); !ONLY ELECTRICITY CAN BE IMPORTED;

@FOR(PERIOD(K): @FOR(OUTPUT(I)|I#NE#6: @SUM(PROCESS(J): A(I,J)*XPARTLOAD(K,J)) - DIS(K,I) - FSLO(K,I) + YI(K,I) = YT(K,I) ));

@FOR(PERIOD(K): @FOR(OUTPUT(I)|I#EQ#6: @SUM(PROCESS(J): A(I,J)*XPARTLOAD(K,J)) - DIS(K,I) - FSLO(K,I) < YT(K,I) ;

@SUM(PROCESS(J): A(I,J)*XPARTLOAD(K,J)) - DIS(K,I) - FSLO(K,I) = YH(K,I) ));

@FOR(PERIOD(K): @FOR(OUTPUT(I)|I #NE#6: YH(K,I) = 0));

@FOR(PERIOD(K): DIS(K,6) = 0; DIS(K,4)=0);

@FOR(PERIOD(K): @FREE(BM));

@FOR(PERIOD(K): @FOR(NOSTORAGE(I): STO(K,I) = 0; FSLO(K,I) = 0));

@FOR(PERIOD(K): @FOR(RM(I): @SUM(PROCESS(J): A(I,J)*XPARTLOAD(K,J)) = YR(K,I)));

@FOR(PERIOD(K): @FOR(PROCESS(J):

!XPARTLOAD(K,J) > PL(J)*X(J) - PL(J)*Z(K,J);

!LET Z(K,J) = X(J)*(SWITCH(K,J));

XPARTLOAD(K,J) < Z(K,J); XPARTLOAD(K,J) > Z(K,J)* PL(J);

Z(K,J) < 1000000*SWITCH(K,J);

Z(K,J) < X(J);

Z(K,J) > X(J) - (1 - SWITCH(K,J))*1000000;

XPARTLOAD(K,J) < SWITCH(K,J)*1000000));

@FOR(BMATRIX: @BIN(SWITCH));

@FOR(PROCESS(J): @FOR(PERIOD(K): X(J)> XPARTLOAD(K,J)));

@FOR(PROCESS(J): @FOR(PERIOD(K): X(J) > Z(K,J)));

@FOR(RMREQ: @FREE(YR));

@FOR(PERIOD(K)| K #LT# 24: @FOR(OUTPUT(I): STO(K+1,I) = STO(K,I) + FSLO(K,I)));

@FOR(PERIOD(K):@FOR(OUTPUT(I): STO(1,I) = STO(24,I) + FSLO(24,I)));

@FOR(DEMAND: @FREE(FSLO));

@FOR(PERIOD(K): @FREE(SALES(K))); @FREE(PROFIT);

@FOR(PERIOD(K): @FOR(STORAGE(I): CAPACITY(I) > STO(K,I)));

@FOR(STORAGE(I): CAPACITY(I) < BST(I)*10000000);

!CONSIDERS THAT DISCHARGED STREAMS CANNOT BE SOLD;

@FOR(PERIOD(K): SALES(K) = @SUM(OUTPUT(I)|I#NE#9 #AND# I#NE#6 : YT(K,I)*P(K,I)) + YH(K,6)*P(K,6) - YI(K,2)*P(K,2)+ @SUM(RM(M):YR(K,M)*P(K,M)) + DIS(K,9)*P(K,9));

PROFIT = (@SUM(PERIOD(K): SALES(K)))*333.33; !CALCULATES FOR ANNUAL PROFIT FROM STREAMS;

CAPCOST = 0.08*(@SUM(PROCESS(J): FC(J)*B(J) + VC(J)*X(J)) + @SUM(STORAGE(I): SFC(I)*BST(I) + SVC(I)*CAPACITY(I)));

!CARBON FOOTPRINT OF SYSTEM;

!DUE TO NET;

@FOR(PERIOD(K): DIS(K,9)*0.0073 = CO2(K)); !CO2 SEQUESTERED PER YEAR;

!DUE TO ELEC IMPORTED;

@FOR(PERIOD(K): YI(K,2)*0.00020 = CF(K)); !carbon footprint of imported electricity is 0.20 kg CO2/kWh;

CARBONFOOTPRINT = @SUM(PERIOD(K): CF(K) - CO2(K))*333.33;

@FREE(CARBONFOOTPRINT);@FREE(FOOTPRINT);

@FOR(PERIOD(K): @FREE(FWATER(K)); @FREE(FHCL(K)));

@FOR(PROCESS(J): X(J) < B(J)*1000000);
